# Highly Pathogenic Avian Influenza A(H5N8) Virus, Democratic Republic of the Congo, 2017

**DOI:** 10.3201/eid2407.172123

**Published:** 2018-07

**Authors:** Augustin T. Twabela, Georges M. Tshilenge, Yoshiro Sakoda, Masatoshi Okamatsu, Ezekiel Bushu, Philippe Kone, Lidewij Wiersma, Gianpiero Zamperin, Alessandra Drago, Bianca Zecchin, Isabella Monne

**Affiliations:** Central Veterinary Laboratory of Kinshasa, Kinshasa I/Gombe, Democratic Republic of the Congo (A.T. Twabela, G.M. Tshilenge);; Hokkaido University, Hokkaido, Japan (A.T. Twabela, Y. Sakoda, M. Okamatsu);; Laboratoire Vétérinaire de Goma, Goma, Democratic Republic of the Congo (E. Bushu);; Food and Agriculture Organization Emergency Center for Transboundary Animal Disease, Kinshasa I (P. Kone);; Food and Agriculture Organization of the United Nations, Rome, Italy (L. Wiersma);; Istituto Zooprofilattico Sperimentale delle Venezie, Legnaro, Italy (G. Zamperin, A. Drago, B. Zecchin, I. Monne)

**Keywords:** avian influenza A(H5N8), Democratic Republic of the Congo, influenza, viruses, respiratory infections, highly pathogenic avian influenza

## Abstract

In 2017, highly pathogenic avian influenza A(H5N8) virus was detected in poultry in the Democratic Republic of the Congo. Whole-genome phylogeny showed the virus clustered with H5N8 clade 2.3.4.4B strains from birds in central and southern Asia. Emergence of this virus in central Africa represents a threat for animal health and food security.

The detection of highly pathogenic avian influenza (HPAI) infections in poultry has greatly increased in the past decades, in particular as a consequence of the spread of the HPAI virus subtype H5, descendent of the H5N1 virus A/goose/Guangdong/1/1996 (Gs/GD), which was detected in China in 1996 (*1*). The evolution of the Gs/GD H5 lineage has resulted in the emergence of multiple clades characterized by distinct antigenic properties and zoonotic potential (*2*). Among them, the HPAI H5 clade 2.3.4.4 has stood out for its concerning ability to reassort and combine with different neuraminidase (NA) subtypes and to spread rapidly to and within multiple continents (*3*).

In late 2016, a reassortant HPAI H5N8 virus (clade 2.3.4.4 group B) began to spread from China (*4*) and the Russian Federation (*5*) to Asia, the Middle East, Europe, and western Africa and for the first time reached central, eastern, and southern Africa. Egypt, Tunisia, and Nigeria reported HPAI H5N8 virus in late autumn 2016, and virus detection continued to occur across Africa in the winter, spring, and summer of 2017 (*6*). This study provides insights from the epidemiologic and viral genome analysis on the outbreaks in the Democratic Republic of the Congo (DRC).

## The Study

**Figure 1:**
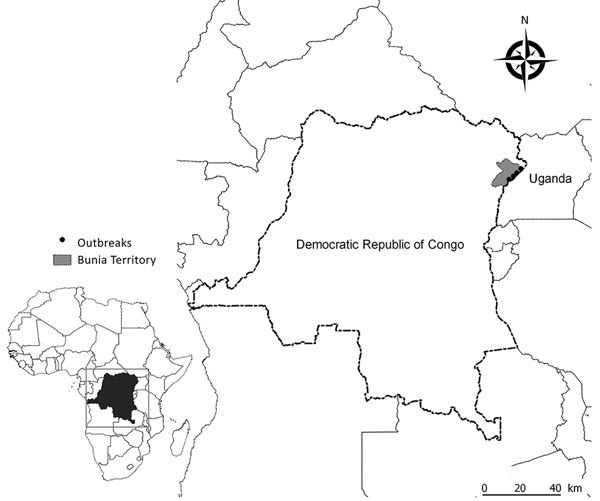
Location of confirmed highly pathogenic avian influenza virus A (H5N8) infection in Bunia territory, on the border with Uganda, Democratic Republic of the Congo, 2017. Inset shows location of Democratic Republic of the Congo in Africa.

In late April 2017, high death rates in domestic chickens and ducks were reported in 4 localities of the Ituri province (Bunia territory) of DRC, which is situated at the edge of Albert Lake between the Rwenzori Mountains and the Republic of Uganda (Figure 1). Because this outbreak followed an HPAI H5N8 outbreak in Uganda in January 2017 (*7**,**8*), this alert led to a strong suspicion of HPAI.

Clinical signs in the affected poultry included prostration, dyspnea, yellowish-colored diarrhea, generalized weakness, torticollis, and, in some cases, recumbency before death. Necropsies on carcasses revealed petechiae, hemorrhage, or both in all organs; hemorrhagic liver with soft consistency; and an empty gizzard with epithelial hemorrhage.

We sampled 22 birds (9 duck carcasses, 12 live ducks, and 1 live chicken) in the 4 infected villages. We collected tracheal and cloacal swabs from living birds showing clinical signs and collected organs including lung, intestine, trachea, and heart from dead birds.

We performed a rapid test for avian influenza virus (AIV) type A detection in the field using the AIV Ag Test Kit (BioNote, Hwaseong-si, South Korea). Of the 22 birds sampled, 6 ducks tested positive with the rapid test; real-time reverse transcription PCR analysis confirmed 11 H5-positive ducks. The Central Veterinary Laboratory of Kinshasa (Kinshasa, DRC) submitted the samples to the World Organisation for Animal Health (OIE) Reference Laboratory and the Food and Agriculture Organization of the United Nations (UN-FAO) Reference Center for Animal Influenza at the Istituto Zooprofilattico Sperimentale delle Venezie (Legnaro, Italy) for confirmatory diagnosis and genetic analysis.

Using an Illumina MiSeq platform (Illumina, San Diego, CA, USA), we obtained whole-genome sequences for 4 viruses selected as being representative of the 4 affected areas in Ituri province (Table; Technical Appendix 1 Table 1. We submitted the full genomes to GenBank (accession nos. MG607401–32) (Table; Technical Appendix 1 Table 1) and used the maximum-likelihood method to generate phylogenetic trees for each gene segment using PhyML 3.1 (http://www.atgc-montpellier.fr/phyml/versions.php).

**Table:** Details of highly pathogenic avian influenza A(H5N8) viruses isolated from birds, Democratic Republic of the Congo, 2017

Date of sample collection	Sampling site	Isolate	GenBank accession no. for hemagglutinin gene
May 14	Tchomia	A/duck/ Democratic Republic of the Congo/17RS882-5/2017	MG607416
May 15	Joo	A/duck/ Democratic Republic of the Congo/17RS882-29/2017	MG607413
May 14	Mahagi	A/duck/ Democratic Republic of the Congo/17RS882-33/2017	MG607414
May 13	Kafe	A/duck/ Democratic Republic of the Congo/17RS882-40/2017	MG607415

**Figure 2:**
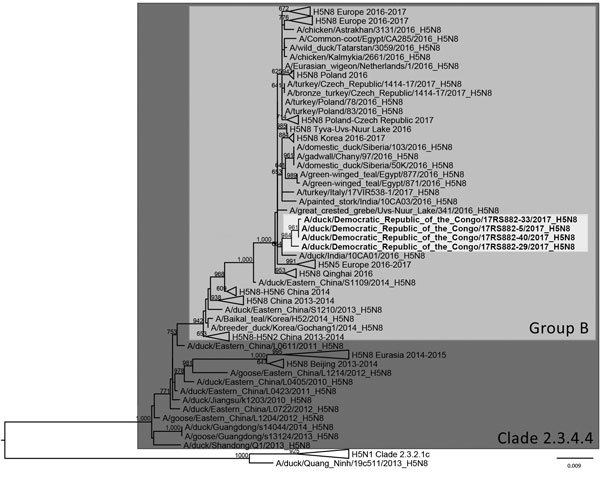
Phylogenetic tree constructed by the maximum-likelihood method of the hemagglutinin gene segment of 4 isolates of highly pathogenic avian influenza A(H5N8) viruses from the Democratic Republic of the Congo (light gray shading) and reference viruses. Bootstrap supports >600/1,000 are indicated above the nodes. Scale bar indicates number of nucleotide substitutions per site.

Among the 4 H5N8 viruses sequenced, A/duck/ Democratic Republic of the Congo/17RS882-5/2017 and A/duck/ Democratic Republic of the Congo/17RS882-40/2017 had identical hemagglutinin (HA) genes; these 2 sequences displayed a similarity of 99.9% with the HA sequences of A/duck/ Democratic Republic of the Congo/17RS882-33/2017 and 99.6% similarity with the HA sequences of A/duck/Democratic Republic of the Congo/17RS882-29/2017. The topology of the phylogenetic tree based on the HA gene segment showed that the H5N8 viruses from DRC belonged to clade 2.3.4.4 group B (*9*) and grouped together with viruses collected in Qinghai, China; southern Russia; and India in 2016. The highest similarity (99.2%) was with an Indian virus (A/duck/India/10CA01/2016) (Figure 2; Technical Appendix 1 Figure 1). For the NA gene, the sequences of the viruses A/duck/ Democratic Republic of the Congo/17RS882-5/2017, A/duck/ Democratic Republic of the Congo/17RS882-33/2017, and A/duck/ Democratic Republic of the Congo/17RS882-40/2017 were identical (100% similarity); these 3 sequences displayed 99.6% similarity with the NA sequence of A/duck/ Democratic Republic of the Congo/17RS882–29/2017(H5N8) (Technical Appendix 1 Figure 2). The phylogenetic trees based on the NA and the internal gene segments (Technical Appendix 1 Figures 1–8), except for the nucleoprotein (NP) gene segment, reflected the same topology of the HA tree, indicating that the H5N8 viruses from DRC were closely related to the virus A/duck/India/10CA01/2016. The topology of the phylogenetic tree based on the NP gene segment (Technical Appendix 1 Figure 4) revealed a different clustering, with the viruses grouped with H5N8 viruses collected from wild birds in Qinghai and southern Russia in 2016. As discussed by Nagarajan et al. (*10*), it is possible that the Indian virus has been involved in a reassortment event that resulted in an NP gene distinct from that described in the Qinghai and southern Russian viruses.

The time to the most recent common ancestor estimated for the HA gene suggested that the H5N8 virus may have reached DRC during March 2016–February 2017 (Technical Appendix 1 Figure 9). However, the paucity of data in the public databases reduces the accuracy of the evolutionary analyses and limits the possibility to reconstruct the early transmission dynamics of the H5N8 virus in DRC.

## Conclusions

Before 2017, no HPAI H5 goose/Guangdong lineage viruses had been reported in DRC. We have attempted to retrace the origin of the H5N8 outbreaks identified in the Ituri province through the evolutionary analysis of viral gene sequences. Considering the close phylogenetic relationship identified between the DRC viruses and those detected in wild and domestic birds in Asia and the overlap of the West Asian–East African flyway with the zones affected by the H5N8 infection, it is reasonable to assume that migratory birds may have been involved in the introduction of the virus in the eastern and central parts of Africa. The inter-African movements of wild birds and the commercial trade between countries could also have favored the spread of AIVs across the region. The outbreaks in Uganda in January 2017 and in DRC in April 2017 could exemplify this scenario because of the close contact between the 2 countries, even though no public information about the genetic characteristics of the Ugandan viruses is available for comparison. According to reports from the DRC veterinary service (www.au-ibar.org/2012-10-01-13.../348-newcastle-disease), different regions of the country have previously reported mortalities in wild and domestic birds; however, these were considered Newcastle disease cases because of the endemic status of this disease in the country and therefore were not investigated further. For the HPAI H5N8 outbreak, the awareness of DRC veterinary services, as well as of the population, was raised following the Uganda HPAI outbreak notification, highlighting the crucial role of sharing information in the control of this transboundary disease. 

Because DRC hosts many sites for residential and migratory wild birds and is considered a stopover point along the West Asian–East African flyway, surveillance in wild and domestic birds should be implemented for early detection of the virus and efficient control of its spread. However, the challenges for the sustainable development of strategies for the effective prevention and control of this disease are vast and deeply ingrained. Investments to overcome infrastructure obstacles hindering the implementation of a true early-warning system are urgently needed to reduce the risk of onward spread of the virus in the region.

Technical Appendix 1 Materials and methods used in the study of highly pathogenic avian influenza A(H5N8), Democratic Republic of the Congo, 2017.

Technical Appendix 2 Hemagglutinin (HA) gene segments of influenza virus strains used for analysis of influenza A(H5N8) virus, Democratic Republic of the Congo, 2017.

## References

[1] Xu X, Subbarao, Cox NJ, Guo Y. Genetic characterization of the pathogenic influenza A/Goose/Guangdong/1/96 (H5N1) virus: similarity of its hemagglutinin gene to those of H5N1 viruses from the 1997 outbreaks in Hong Kong. Virology. 1999;261:15–9. 10.1006/viro.1999.9820 10484749

[2] World Health Organization/World Organisation for Animal Health/Food and Agriculture Organization (WHO/OIE/FAO) H5N1 Evolution Working Group. Revised and updated nomenclature for highly pathogenic avian influenza A (H5N1) viruses. Influenza Other Respi Viruses. 2014;8:384–8. 10.1111/irv.12230 24483237 PMC4181488

[3] Yu Y, Zhang Z, Li H, Wang X, Li B, Ren X, et al. Biological characterizations of H5Nx avian influenza viruses embodying different neuraminidases. Front Microbiol. 2017;8:1084. 10.3389/fmicb.2017.01084 28659898 PMC5469879

[4] Li M, Liu H, Bi Y, Sun J, Wong G, Liu D, et al. Highly pathogenic avian influenza A(H5N8) virus in wild migratory birds, Qinghai Lake, China. Emerg Infect Dis. 2017;23:637–41. 10.3201/eid2304.161866 28169827 PMC5367427

[5] Lee DH, Sharshov K, Swayne DE, Kurskaya O, Sobolev I, Kabilov M, et al. Novel reassortant clade 2.3.4.4 avian influenza A(H5N8) virus in wild aquatic birds, Russia, 2016. Emerg Infect Dis. 2017;23:359–60. 10.3201/eid2302.161252 27875109 PMC5324796

[6] World Organisation for Animal Health. Immediate notifications and follow-up reports of highly pathogenic avian influenza (types H5 and H7). 2017 [cited 2017 Jun 30]. http://www.oie.int/en/animal-health-in-the-world/update-on-avian-influenza/2017

[7] Centers for Disease Control and Prevention. Global health protection and security. 2017 [cited 2017 Aug 31]. https://www.cdc.gov/globalhealth/healthprotection/fieldupdates/summer-2017/uganda-avian-influenza.html

[8] World Organisation for Animal Health. Avian influenza country report. 2017 [cited 2017 Jun 30]. http://www.oie.int/wahis_2/public%5C.%5Ctemp%5Creports/en_fup_0000024015_20170615_135858.pdf

[9] Smith GJ, Donis RO; World Health Organization/World Organisation for Animal Health/Food and Agriculture Organization (WHO/OIE/FAO) H5 Evolution Working Group. Nomenclature updates resulting from the evolution of avian influenza A(H5) virus clades 2.1.3.2a, 2.2.1, and 2.3.4 during 2013-2014. Influenza Other Respi Viruses. 2015;9:271–6. 10.1111/irv.12324 25966311 PMC4548997

[10] Nagarajan S, Kumar M, Murugkar HV, Tripathi S, Shukla S, Agarwal S, et al. Novel reassortant highly pathogenic avian influenza (H5N8) virus in zoos, India. Emerg Infect Dis. 2017;23:717–9. 10.3201/eid2304.161886 28117031 PMC5367432

