# Supplementary material for: Highly Pathogenic Avian Influenza A(H5N8) Virus, Democratic Republic of the Congo, 2017
Source: Emerg Infect Dis. 2018 Jul;24(7):1371–4. doi: 10.3201/eid2407.172123 (PMC6038760; doi:10.3201/eid2407.172123)
Supplement: Technical Appendix 1 — Materials and methods used in the study of highly pathogenic avian influenza A(H5N8), Democratic Republic of the Congo, 2017. [file 17-2123-Techapp-s1.pdf]

Article DOI: https://doi.org/10.3201/eid2407.172123

Highly Pathogenic Avian Influenza A(H5N8) Virus, Democratic Republic of the Congo, 2017

Technical Appendix

Materials and Methods

Genome Amplification and Sequencing

We purified total RNA from 4 HPAI H5N8-positive clinical samples, collected in the Democratic Republic of the Congo (DRC), using the Nucleospin RNA kit (Macherey-Nagel, Düren, Germany). We amplified whole influenza A virus genomes using the SuperScript III One-Step RT-PCR system with Platinum Taq High Fidelity (Invitrogen, Carlsbad, CA, USA) (*1*). Following the manufacturer's instructions, we obtained sequencing libraries using the Nextera DNA XT sample preparation kit (Illumina, San Diego, CA, USA) and quantified them using the Qubit dsDNA High Sensitivity kit (Invitrogen). We determined the average fragment length using the Agilent High Sensitivity Bioanalyzer Kit (Agilent Technologies, Santa Clara, CA, USA). We sequenced the indexed libraries in multiplex for 250 bp paired-end on Illumina MiSeq.

High-Throughput Sequencing Data Analysis

We assessed read quality using FastQC v0.11.2 (https://www.bioinformatics.babraham.ac.uk/projects/fastqc). We filtered the raw data by removing reads with more than 10% of undetermined (N) bases, reads with more than 100 bases with Q score below 7, and duplicated paired-end reads. We clipped the remaining reads from Illumina Nextera XT adaptors with scythe v0.991 (https://github.com/vsbuffalo/scythe) and trimmed them with sickle v1.33 (https://github.com/najoshi/sickle). We aligned high-quality reads against a reference genome using BWA v0.7.12 (*2*). We processed alignments with Picard-tools v2.1.0 (http://picard.sourceforge.net) and GATK v3.5 (*3*–*5*). We called single nucleotide

polymorphisms were called using LoFreq v2.1.2 (*6*) and used the outputs to generate the consensus sequences.

Phylogenetic Analyses

We aligned consensus sequences of the complete genome of the 4 samples from DRC (Technical Appendix 1 Table 1) using MAFFT v7 (*7*) and compared them with the most related sequences available in the Global Initiative on Sharing All Influenza Data (GISAID) database (https://www.gisaid.org) (Technical Appendix 2, https://wwwnc.cdc.gov/EID/article/24/7/17-2123-Techapp2.xlsx). In detail, for each gene segment, we aligned the sequences of the H5N8 viruses from DRC with all the H5 sequences belonging to clade 2.3.4.4 group B available in GISAID at the time of writing and with the 50 most related sequences resulted from a BLAST search (https://blast.ncbi.nlm.nih.gov/Blast). We obtained maximum-likelihood phylogenetic trees using the best-fit general time-reversible model of nucleotide substitution with gamma-distributed rate variation among sites (with 4 rate categories, $\Gamma4$) and a heuristic SPR branch-swapping search (*8*) available in PhyML v3.1 (http://www.atgc-montpellier.fr/phyml/versions.php). We performed 1,000 bootstrap replicates. Phylogenetic trees were visualized using FigTree v1.4.2 (http://tree.bio.ed.ac.uk/software/figtree/) (Technical Appendix 1 Figures 1–9).

Estimation of Time to the Most Recent Common Ancestor

Using BEAST v.1.8.4 software (*9*), we estimated the tMRCA of the HPAI H5N8 identified in the DRC for the HA gene segment. We selected a HKY85 + Γ_4 nucleotide substitution model with 2 data partitions reflecting codon positions (1st + 2nd positions, 3rd position) with base frequency unlinked across all codon position (SRD06 substitution model). We adopted a relaxed uncorrelated lognormal molecular clock and constant population size as the tree prior. We used Markov chain Monte Carlo (MCMC) and chain lengths of 100 million iterations to achieve convergence. We used TreeAnnotator v1.8.4 to summarize the maximum clade credibility (MCC) phylogenetic tree from the posterior distribution of trees, after the removal of a burn-in of 10% of the samples. We used FigTree v1.4.2 (http://tree.bio.ed.ac.uk/software/figtree/) to visualize the MCC tree (Technical Appendix 1 Figure 9).

References

1. Zhou B, Donnelly ME, Scholes DT, St. George K, Hatta M, Kawaoka Y, et al. Single-reaction genomic amplification accelerates sequencing and vaccine production for classical and swine origin human influenza A viruses. J Virol. 2009;83:10309–13. PubMed http://dx.doi.org/10.1128/JVI.01109-09

2. Li H, Durbin R. Fast and accurate long-read alignment with Burrows-Wheeler transform. Bioinformatics. 2010;26:589–95. PubMed http://dx.doi.org/10.1093/bioinformatics/btp698

3. McKenna A, Hanna M, Banks E, Sivachenko A, Cibulskis K, Kernytsky A, et al. The Genome Analysis Toolkit: a MapReduce framework for analyzing next-generation DNA sequencing data. Genome Res. 2010;20:1297–303. PubMed http://dx.doi.org/10.1101/gr.107524.110

4. DePristo MA, Banks E, Poplin R, Garimella KV, Maguire JR, Hartl C, et al. A framework for variation discovery and genotyping using next-generation DNA sequencing data. Nat Genet. 2011;43:491–8. PubMed http://dx.doi.org/10.1038/ng.806

5. Van der Auwera GA, Carneiro MO, Hartl C, Poplin R, Del Angel G, Levy-Moonshine A, et al. From FastQ data to high confidence variant calls: the Genome Analysis Toolkit best practices pipeline. Curr Protoc Bioinformatics. 2013;43:11.10.1–33. http://dx.doi.org/10.1002/0471250953.bi1110s43

6. Wilm A, Aw PP, Bertrand D, Yeo GH, Ong SH, Wong CH, et al. LoFreq: a sequence-quality aware, ultra-sensitive variant caller for uncovering cell-population heterogeneity from high-throughput sequencing datasets. Nucleic Acids Res. 2012;40:11189–201. PubMed http://dx.doi.org/10.1093/nar/gks918

7. Katoh K, Standley DM. MAFFT multiple sequence alignment software version 7: improvements in performance and usability. Mol Biol Evol. 2013;30:772–80. PubMed http://dx.doi.org/10.1093/molbev/mst010

8. Guindon S, Gascuel O. A simple, fast, and accurate algorithm to estimate large phylogenies by maximum likelihood. Syst Biol. 2003;52:696–704. PubMed http://dx.doi.org/10.1080/10635150390235520

9. Drummond AJ, Rambaut A. BEAST: Bayesian evolutionary analysis by sampling trees. BMC Evol Biol. 2007;7:214. PubMed http://dx.doi.org/10.1186/1471-2148-7-214

Technical Appendix Table. Epidemiologic information and gene segments accession numbers of the H5N8 viruses characterized in this study, Democratic Republic of the Congo, May 2017

Date of sample collection	Sampling site	Isolate	Accession number*
May 14	TCHOMIA	A/duck/Democratic Republic of the Congo/17RS882–5/2017 (H5N8)	MG607404 for PB2 gene; MG607408 for PB1 gene; MG607412 for PA gene; MG607420 for NP gene; MG607424 for NA gene; MG607428 for MP gene; MG607432 for NS gene
May 15	JOO	A/duck/ Democratic Republic of the Congo/17RS882–29/2017 (H5N8)	MG607401 for PB2 gene; MG607404 for PB1 gene; MG607409 for PA gene; MG607417 for NP gene; MG607421 for NP gene; MG607425 for MP gene; MG607429 for NS gene
May 14	MAHAGI	A/duck/ Democratic Republic of the Congo/17RS882–33/2017 (H5N8)	MG607402 for PB2 gene; MG607406 for PB1 gene; MG607410 for PA gene; MG607418 for NP gene; MG607422 for NA gene; MG607426 for MP gene; MG607430 for NS gene
May 13	KAFE	A/duck/ Democratic Republic of the Congo/17RS882–40/2017 (H5N8)	MG607403 for PB2 gene; MG607407 for PB1 gene; MG607411 for PA gene; MG607419 for NP gene; MG607423 for NA gene; MG607427 for MP gene; MG607431 for NS gene

*HA, hemagglutinin; MP, matrix protein; NA, neuraminidase; NP, nucleoprotein; NS, nonstructural protein; PA, polymerase acidic protein; PB1, polymerase basic protein 1; PB2, polymerase basic protein 2

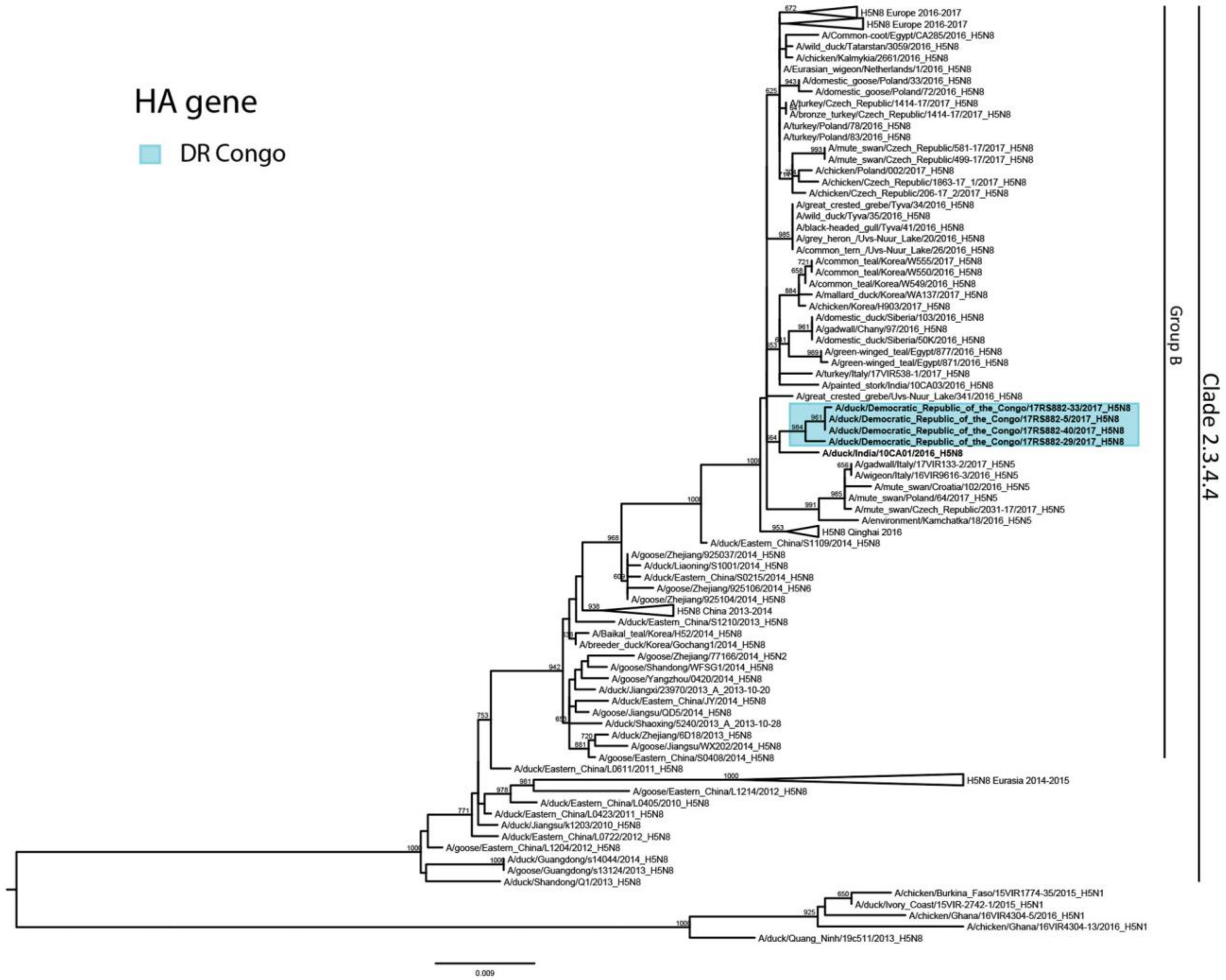

Technical Appendix Figure 1. Phylogenetic trees constructed by the maximum-likelihood method of the hemagglutinin (HA) gene segment of the 4 isolates of highly pathogenic avian influenza virus A(H5N8) from the Democratic Republic of the Congo in May 2017. Viruses analyzed in this study are highlighted in red. Bootstrap supports higher than 600/1000 are indicated above the nodes. Scale bar indicates number of nucleotide substitutions per site.

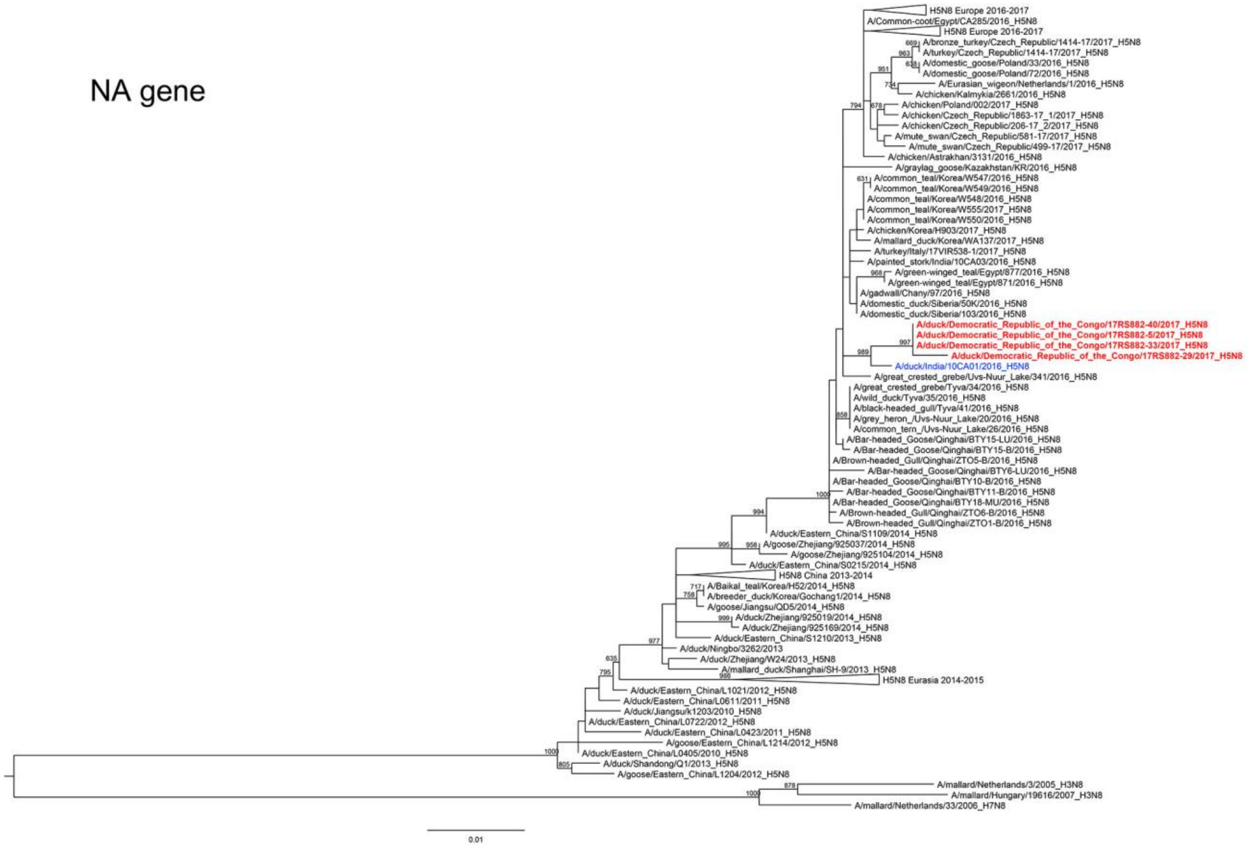

Technical Appendix Figure 2. Maximum-likelihood phylogenetic tree of the neuraminidase (NA) gene. The H5N8 viruses from the Democratic Republic of the Congo are marked in red. Bootstrap supports higher than 600/1000 are indicated next to the nodes. Scale bar indicates number of nucleotide substitutions per site.

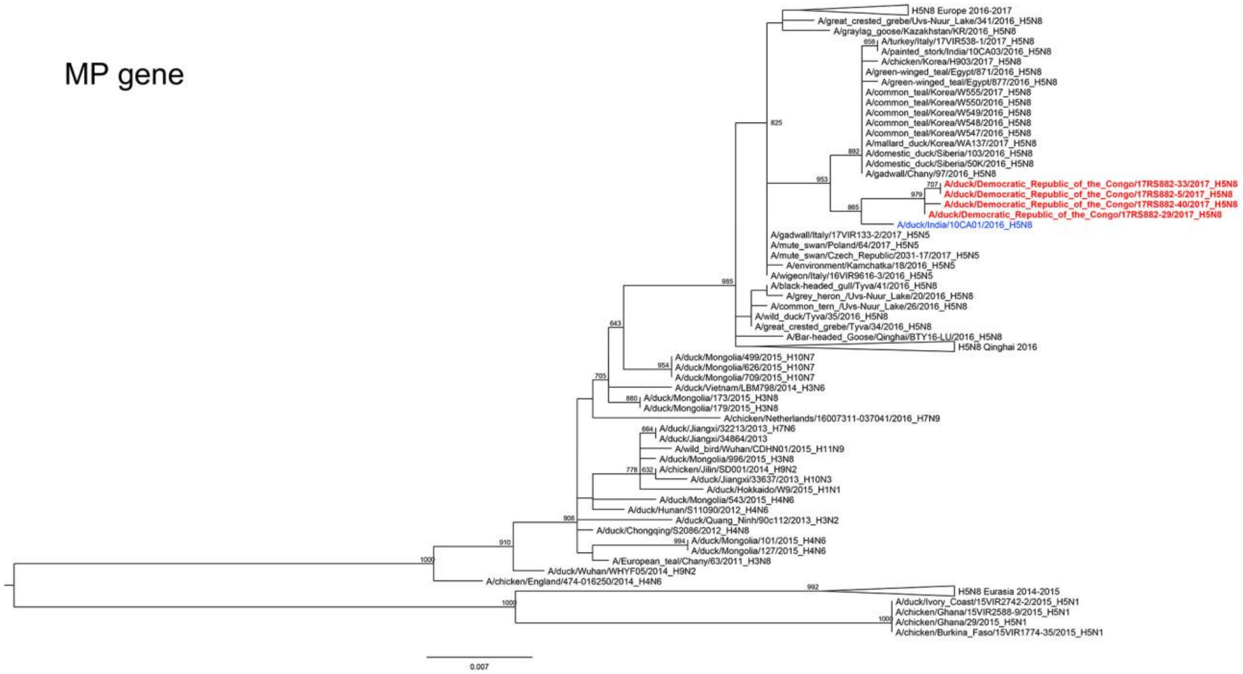

Technical Appendix Figure 3. Maximum-likelihood phylogenetic tree of the matrix protein (MP) gene. The H5N8 viruses from the Democratic Republic of the Congo are marked in red. Bootstrap supports higher than 600/1000 are indicated next to the nodes. Scale bar indicates number of nucleotide substitutions per site.

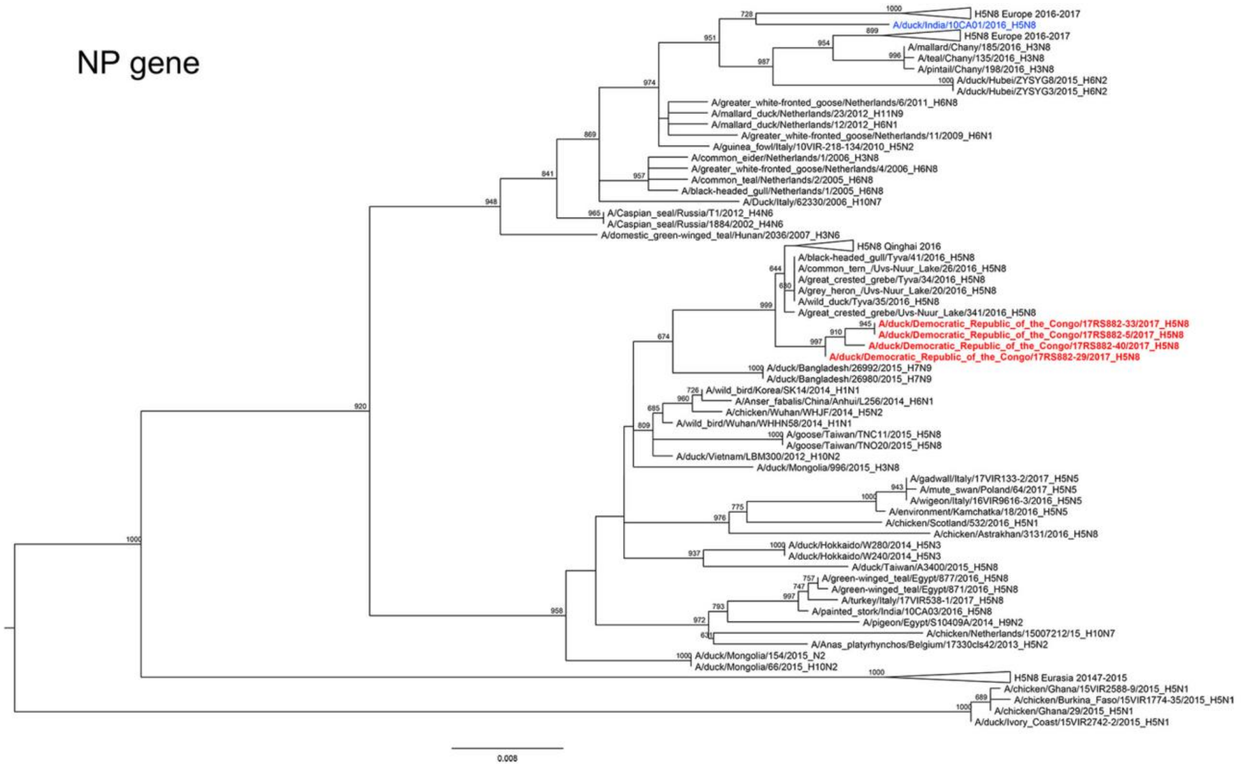

Technical Appendix Figure 4. Maximum-likelihood phylogenetic tree of the nucleoprotein (NP) gene. The H5N8 viruses from the Democratic Republic of the Congo are marked in red. Bootstrap supports higher than 600/1000 are indicated next to the nodes. Scale bar indicates number of nucleotide substitutions per site.

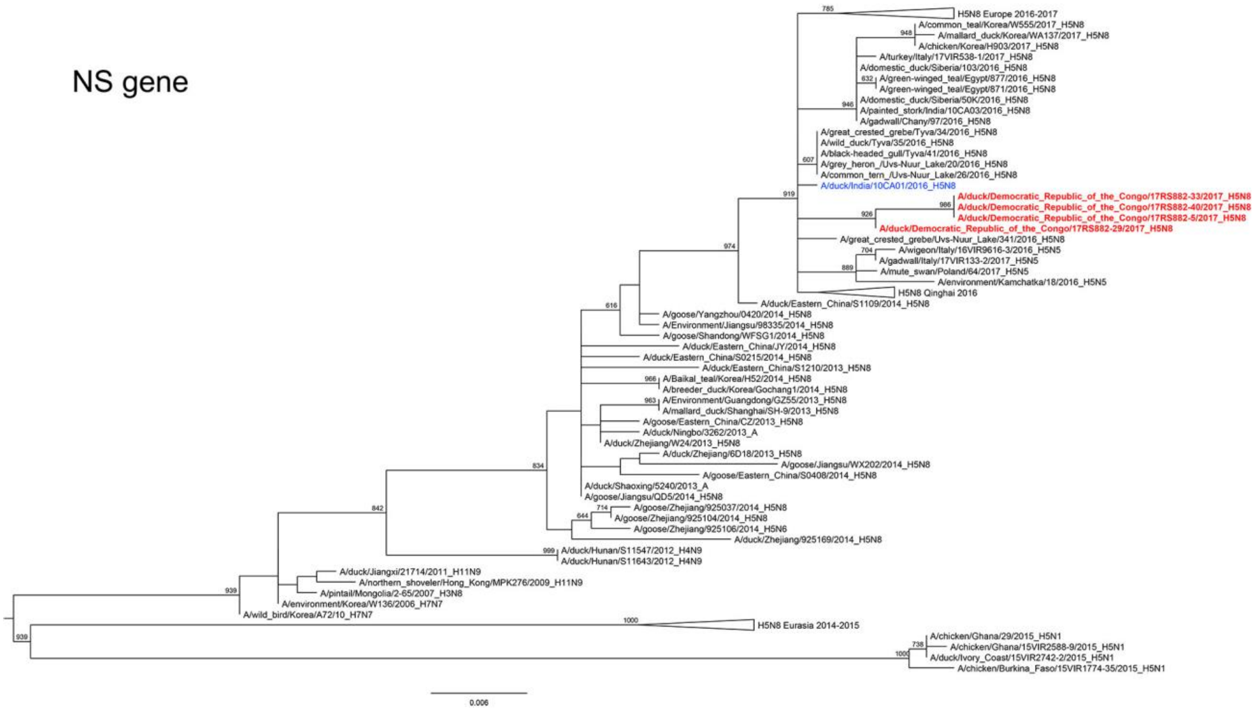

Technical Appendix Figure 5. Maximum-likelihood phylogenetic tree of the nonstructural protein (NS) gene. The H5N8 viruses from the Democratic Republic of the Congo are marked in red. Bootstrap supports higher than 600/1000 are indicated next to the nodes. Scale bar indicates number of nucleotide substitutions per site.

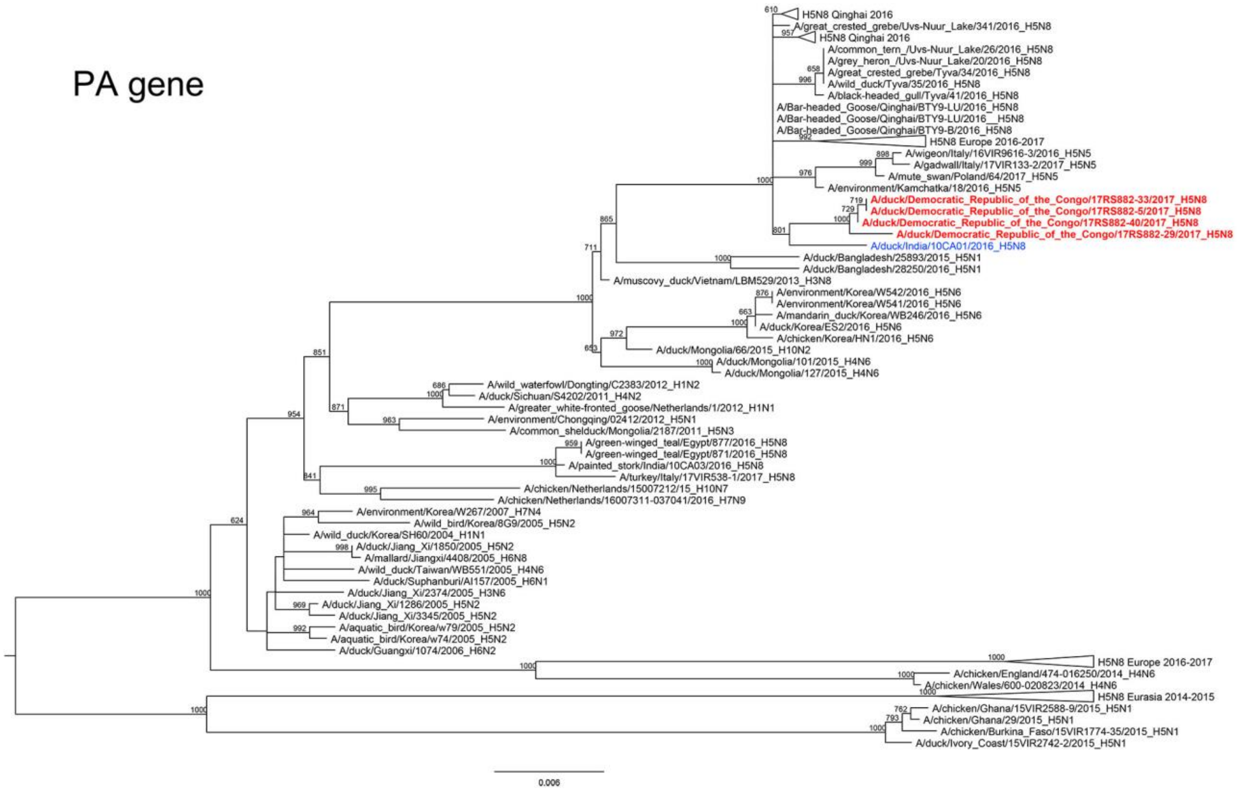

Technical Appendix Figure 6. Maximum-likelihood phylogenetic tree of the polymerase acidic protein (PA) gene. The H5N8 viruses from the Democratic Republic of the Congo are marked in red. Bootstrap supports higher than 600/1000 are indicated next to the nodes. Scale bar indicates number of nucleotide substitutions per site.

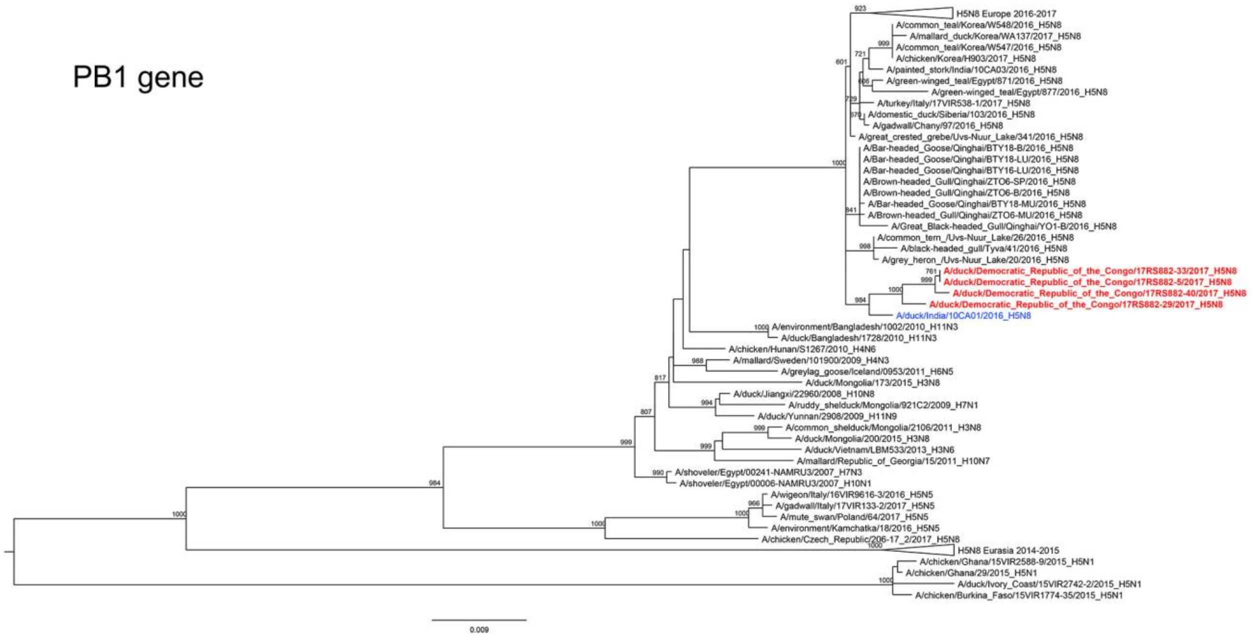

Technical Appendix Figure 7. Maximum-likelihood phylogenetic tree of the polymerase basic protein 1 (PB1) gene. The H5N8 viruses from the Democratic Republic of the Congo are marked in red. Bootstrap supports higher than 600/1000 are indicated next to the nodes. Scale bar indicates number of nucleotide substitutions per site.

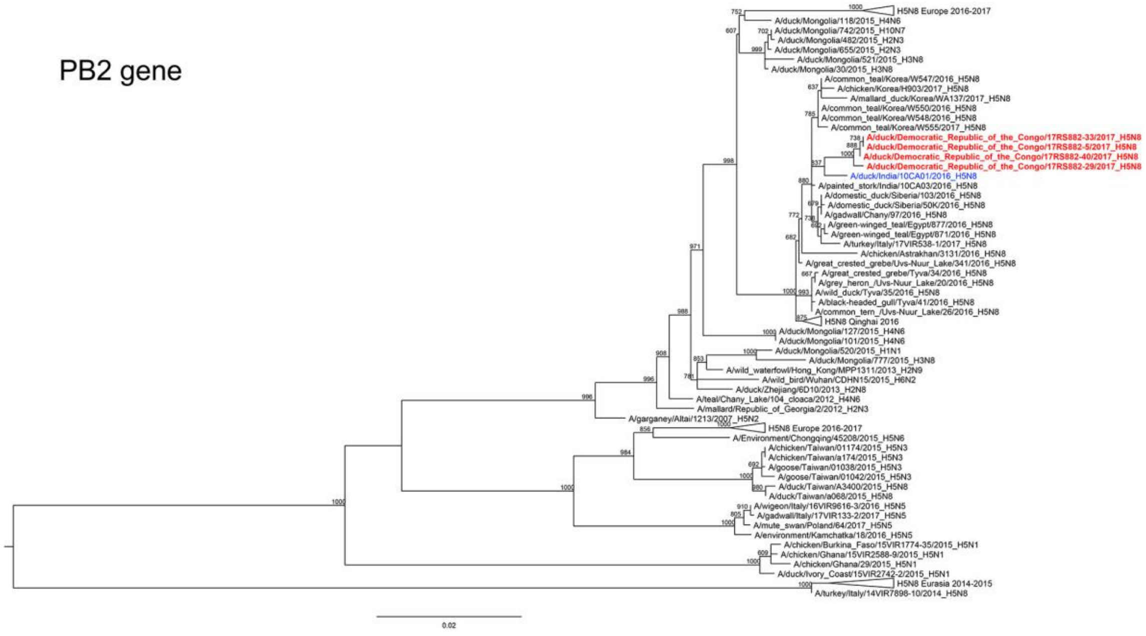

Technical Appendix Figure 8. Maximum-likelihood phylogenetic tree of the polymerase basic protein 2 (PB2) gene. The H5N8 viruses from the Democratic Republic of the Congo are marked in red. Bootstrap supports higher than 600/1000 are indicated next to the nodes. Scale bar indicates number of nucleotide substitutions per site.

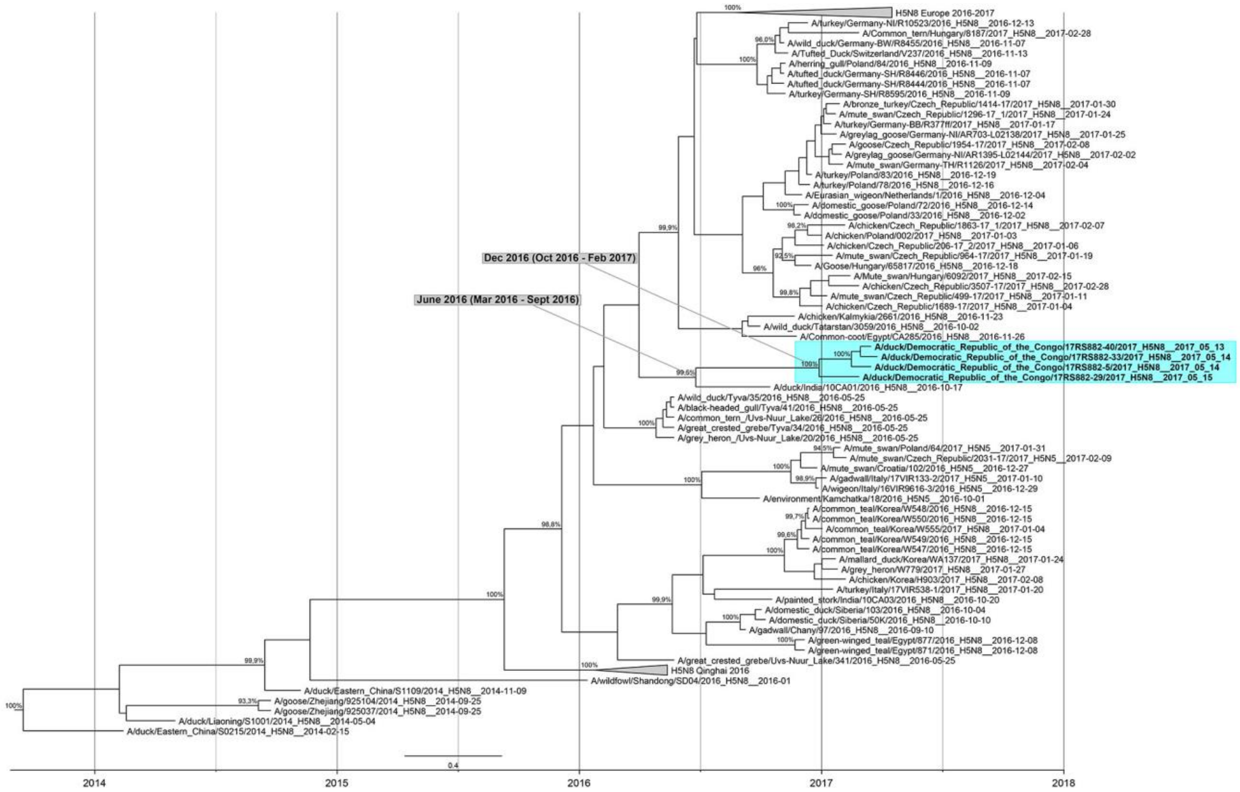

Technical Appendix Figure 9. Maximum clade credibility phylogenetic tree of the hemagglutinin (HA) gene. The H5N8 viruses from the Democratic Republic of the Congo are highlighted in the light blue box. Posterior probability values higher than 90% are indicated next to the nodes. The mean time to the most recent common ancestor (tMRCA) and the 95% highest posterior density intervals of the relevant nodes are indicated in the gray boxes.
